# Accuracy of Total Hip Arthroplasty Templating Using Set Calibration Magnifications

**DOI:** 10.7759/cureus.34883

**Published:** 2023-02-12

**Authors:** Benjamin Schapira, Suroosh Madanipour, Farhad Iranpour, Padmanabhan Subramanian

**Affiliations:** 1 Trauma and Orthopaedics, Royal Free London NHS Foundation Trust, London, GBR; 2 Trauma and Orthopaedics, Princess Alexandra Hospital, Harlow, GBR

**Keywords:** preoperative templating, calibration, magnification, tha, total hip arthroplasty: tha

## Abstract

Background

Templating for total hip arthroplasty has been adopted over recent decades as a reliable and accurate method for pre-operative planning. The use of calibration markers for this process provides a recognised benefit at the expense of cost, availability and error. Many surgeons use a set magnification of 118% to account for calibration errors when templating total hip arthroplasty. This study aims to assess the accuracy of templating with standardised magnifications and assess the effect of BMI on templating accuracy.

Materials and methods

A retrospective analysis was performed using a single-surgeon series of 119 consecutive total hip arthroplasties. Anteroposterior radiographs were taken pre- or post-operatively without calibration hardware. Pre-operatively, the total hip arthroplasty was templated on TraumaCad (BrainLab Inc, Westchester, IL) using either 118% or 119% calibration magnification. Post-operative magnification was calibrated using the known femoral head diameter. Templated and implanted prostheses were compared for size.

Results

At 118%, 61.1% of cups matched those templated with 96.3% of cups within two sizes. At 119%, 52.5% of cups used matched their templates with 100% within two sizes. There was no significant difference between 118% and 119% cup size prediction (p=0.49). A trend was noticed in increasing magnification error with increasing BMI. However, BMI had no significant effect on the accuracy of templating cup size within two cup sizes (p=0.58).

*Conclusion*.

Templating acetabular cups using a set magnification of 118% or 119% yields accurate results and provides a reliable method to template without calibration equipment. Whilst BMI can affect magnification error, this has no significant effect on the accuracy of implanted cups and stems within two sizes.

## Introduction

Templating for total hip arthroplasty (THA) has been adopted over recent decades as a reliable and accurate method for pre-operative planning [1]. Benefits have been noted in improved anatomical planning [2,3], operative risk assessment [4,5] and estimation of implant choice [6,7]; with subsequent benefits for cost and implant longevity [8,9].

Despite increasing popularity, studies show relatively poor uptake for templating THAs. In 2011, one study found that amongst NHS trusts in South East England, only 14/28 (50%) had access to digital templating software, with just one of these trusts offering training [10]. None of the trusts regularly templated or calibrated for magnification in pre-operative planning. Despite these figures, templating has been proven to enhance surgical accuracy for all levels of training, up to the most experienced surgeons [11].

Current techniques involve expensive external calibration markers (ECMs) which often present great variation from true magnification, amounting to as much as 6.8% error or a difference of +/- 3mm in acetabular cup sizes [7,12]. These results insist markers cannot accurately predict the true magnification in pre-operative templating [6].

Given the inaccuracies and ease of misplacing ECMs, some surgeons have opted to template without such equipment and elect a standard magnification for all templating (set magnification), the proponents of which claim greater estimation of magnification, reduced cost of calibration hardware and improved templating accuracy [6,13]. The primary objectives of this study were to identify whether set magnifications for digital templating could accurately enable surgeons to estimate prosthesis size. We also aim to investigate the effects of BMI on magnification and subsequent templating accuracy. This article was previously presented as a meeting abstract at the 2021 EFORT (European Federation of National Associations of Orthopaedics and Traumatology) annual virtual congress.

## Materials and methods

A retrospective analysis was performed in accordance with the STROBE (Strengthening the Reporting of Observational Studies in Epidemiology) and STARD (Standards for Reporting Diagnostic Accuracy) checklists, using a single-surgeon series of 136 consecutive THAs from April 2019-November 2020. All cases took place within the same centre during the senior-most consultants’ tenure. The mean age of patients was 70.1 years (standard deviation 11.47 years) and 56.3% of patients were female. This study followed the usual protocols of investigation, intervention and retrospective analysis of our trust's Quality Governance Team with respect to ethical approval and patient consent. All data was kept anonymised and followed in accordance with the 1975 declaration of Helsinki and its 2008 revision. Indications for primary THA included osteoarthritis, rheumatoid arthritis, avascular necrosis, dysplasia and fractured neck of the femur. Exclusion criteria included revision surgery, absence of pre-operative template and where pre-operative BMI was not recorded.

Anteroposterior (AP) pelvic radiographs were taken pre-operatively and 0-2 days post-operatively using no calibration hardware (calibration balls or mats) with a standardised radiographic protocol followed in our trust. All radiographs conformed to the same protocol using the Samsung XGEO GC80 series (Samsung Electronics Co., Ltd, Yeongtong-gu, Suwon, South Korea). Patients were positioned supine with feet internally rotated to optimise symmetry. The tube-detector distance was routinely set to 100cm with the radiographic beam aimed at the level of the greater trochanters. BMI was recorded as part of the pre-operative assessment. 

**Figure 1:**
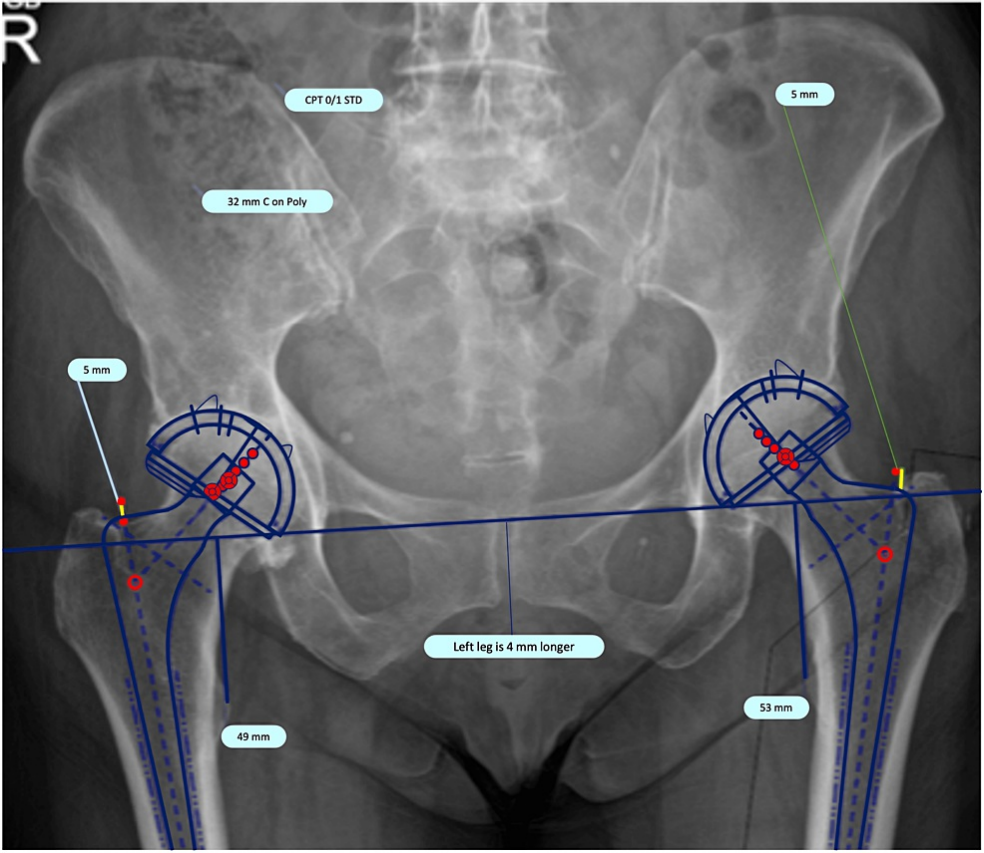
Pre-operative anteroposterior pelvic radiograph of a 45-year-old female on TraumaCad software calibrated to 118% magnification demonstrating templating technique. TraumaCad (BrainLab Inc, Westchester, IL)

The predominant prostheses used in templating were the CPT and Trilogy systems (Zimmer, Warsaw, IN, US) with a minority using the G7 dual-mobility systems (Zimmer) in those with significant pre-operative risk factors for dislocation. All cases used CPT femoral stems. Templating aimed to restore the patient’s native anatomy using the acetabular teardrop as a reference point for cup placement. The femoral polished tapered stem component was sized to allow for appropriate restoration of the patient’s native offset, leg length and caput-collum-diaphyseal angle with a 2mm cement mantle. Planned acetabular cup size, stem size and offset were recorded in retrospect from the template (Figure 1). A posterior approach was utilised using a standard incision. Intra-operatively, the template was used as a guide and the surgeon chose the final prosthesis with complete autonomy.

Pre-operatively, the THA was templated on TraumaCad (BrainLab Inc, Westchester, IL) software using either 118% or 119% calibration magnification (unless the contralateral side had a THA with a known femoral head diameter). Pre-operative template magnification was chosen at random. All cases were templated and operated upon by a single consultant hip and knee arthroplasty surgeon.

**Figure 2:**
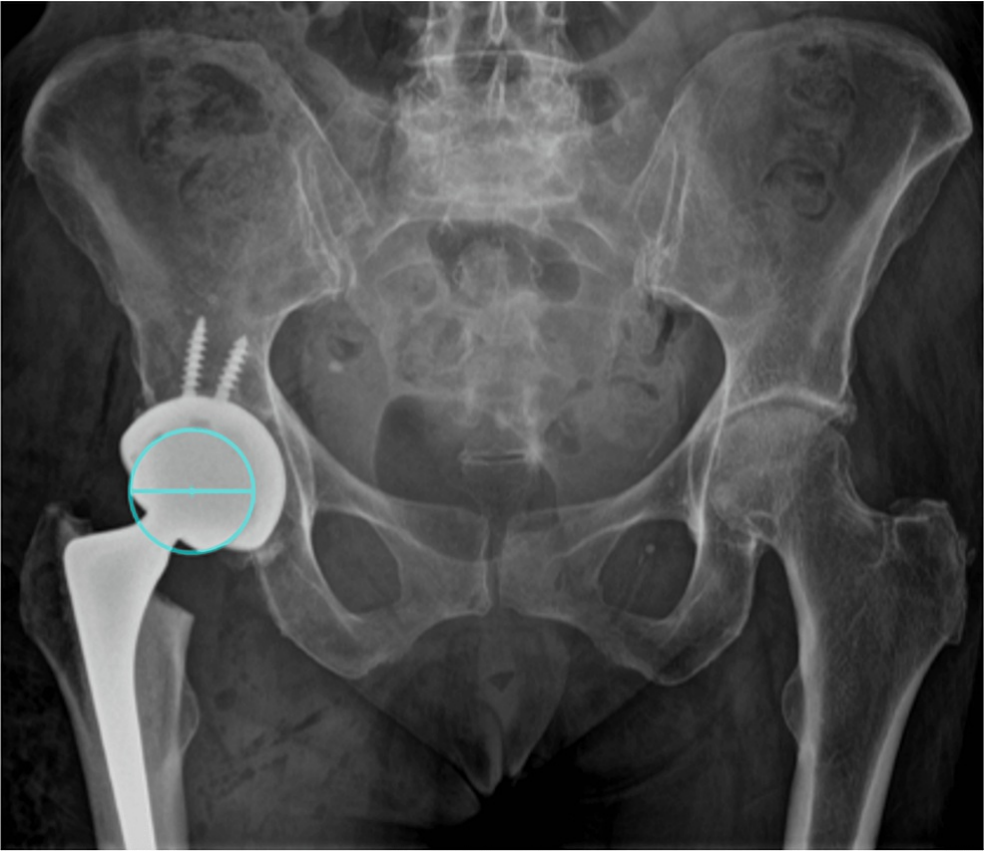
Post-operative anteroposterior pelvic radiograph visualised of a 45-year-old female on TraumaCad templating software for calibration of magnification using the known size of the femoral head.

Post-operative radiographs were reviewed on TraumaCad by a single-blinded observer who was not involved with initial templating. Magnification was calibrated using the known diameter of the femoral head (true magnification) (Figure 2). Most cases with Trilogy acetabular cups used 36mm femoral heads, with three cases using 32mm and where a dual mobility component was used, a 28mm liner head was used (four cases). The true cup size and stem length were recorded from the operative notes and compared with the template choice. The true magnification was compared with the set magnification used pre-operatively to calculate the magnification error (pre-operative set magnification - post-operative true magnification).

Statistical analysis

Statistical analyses were performed on IBM SPSS Statistics Software version 26 (IBM Corp, Armonk, NY, US). The chi-square and Fisher’s exact test were used to compare categorical data. Independent t-tests and Mann-Whitney U tests were used to compare continuous results. A p-value of <0.05 was considered statistically significant.

## Results

In total, 136 THA cases were reviewed, of which 12 revision cases and five further cases with no pre-operative template were excluded. After all exclusions, 119 cases remained for analysis. 

The median cup size templated was 54mm (range 46-64mm), the median stem templated was CPT size 1 (range 0-3) and the median modular neck option templated was the extended size (+5mm additional offset). The median cup size used was 54mm (range 44-66mm) using Trilogy in 96 cases and G7 in 23 and the median stem size used was CPT size 1 (range 0-4). 

Retrospective analysis of all pre-operative radiographs found 54 cases were calibrated at 118% and 40 cases at 119% magnification. In cases templated to 118%, post-operative radiographic magnification matched the pre-operative template in 42.6% of cases. This increased to 74.1% within a 2% error. In cases templated with 119% magnification, post-operative radiographic magnification matched the pre-operative template in 12.5% of cases and 70% within 2% error.

**Figure 3:**
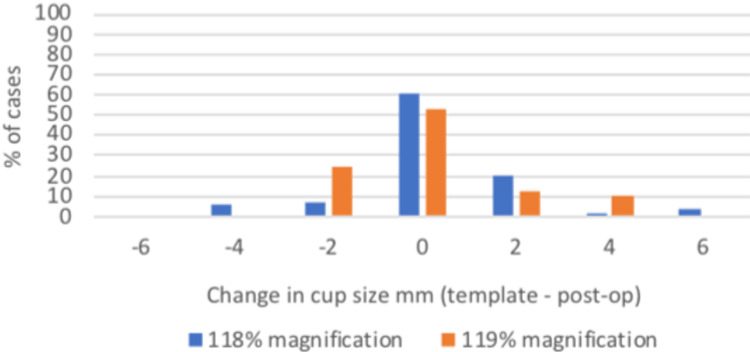
A graph presenting the difference in templated and implanted acetabular cup size when templating at 118% and 119% magnification (2mm = 1 cup size).

At 118% magnification, 61.11% of cases used the same cup size templated with 96.3% within two sizes of the template. At 119% magnification, 52.5% of cases used the same cup size templated with 100% within two sizes of the template (Figure 3). There was no significant difference between 118% and 119% cup size prediction (p=0.49). 

**Figure 4:**
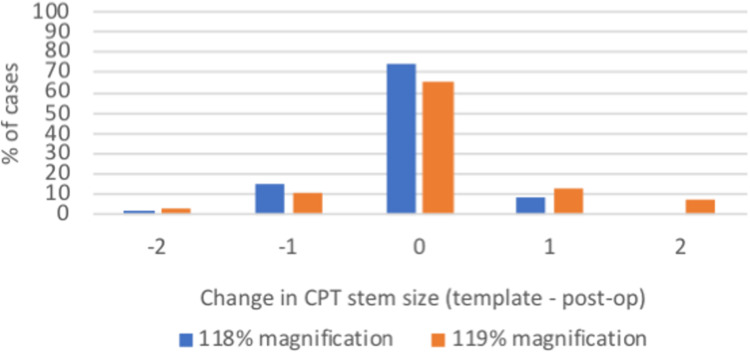
A graph presenting the difference in templated and implanted femoral stems when templating at 118% and 119% magnification. At both template magnifications, all stems implanted were accurate to within two sizes.

The implanted femoral stem size matched the template size at 74.1% at 118% template magnification and 65.8% at 119% with both increasing to 100% within two stem sizes (Figure 4). 

**Figure 5:**
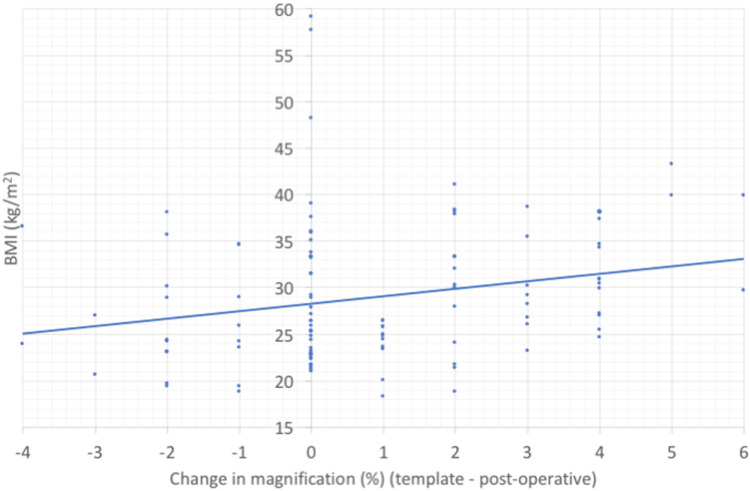
A plot of the difference in calibration magnification between pre and post-operative radiographs against patient BMI. The line of best fit represents the relationship between increasing BMI and increasing magnification error.

**Figure 6:**
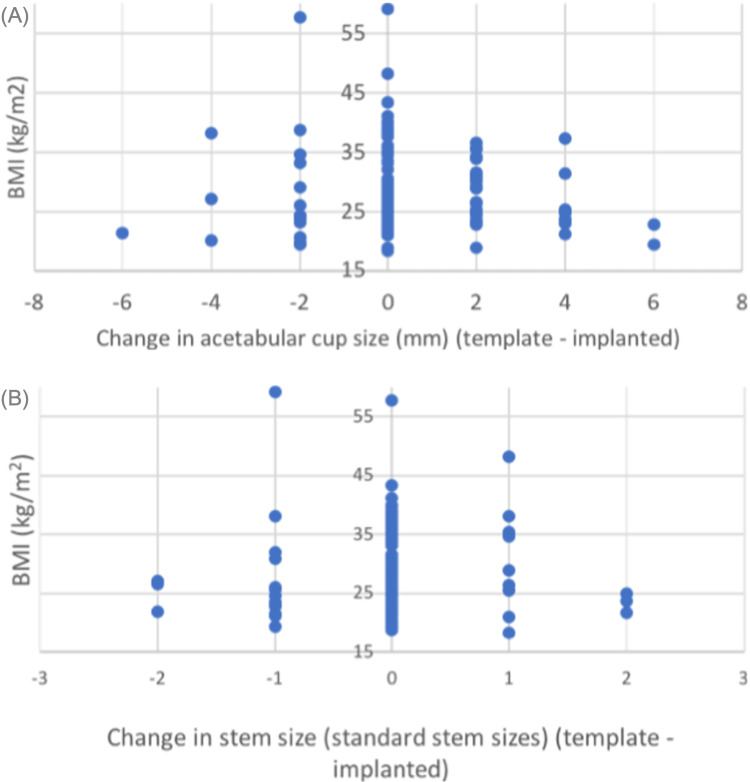
(A) A plot demonstrating the change in acetabular cup size with BMI across all cases (one cup size = 2mm). (B) A plot demonstrating the change in femoral stem size with BMI across all cases.

In total, seven cases did not have pre-operative BMI recorded. The mean BMI in our study was 30.42kg/m2 with 22 cases (20.75%) above 35kg/m2 and five cases (4.72%) above 40kg/m2. A trend was noticed in increasing magnification error with increasing BMI. However, BMI had no significant effect on the accuracy of templating the acetabulum within two cup sizes (p=0.58) (Figure 5). Where implanted cup or stem size differed by more than two sizes from the template, BMI ranged from 19.5-22.8 kg/m2 (Figure 6). 

## Discussion

To our knowledge, this is the very first study to research the accuracy of different set magnifications in digital THA templating. Our results proved high accuracy and low magnification error at both 118% and 119% for both acetabular cup and femoral stem template predictions. These suggest templating at a set magnification without ECMs will provide reliable and accurate pre-operative planning.

Very few studies investigate the accuracy of templating without magnification markers. One study presented exact cup size selection in 86% and 64% in stem selection when set magnification was estimated at 116% [14]. Si et al. reported exact cup prediction in 43.3% and stem prediction in 40% of cases, improving to 95.56% and 93.33% within two sizes respectively, without an ECM [5]. Our results compare favourably with studies reporting calibration errors of 1.42% using a set magnification of 121% compared with errors as high as 2.55% with ECMs [15]. At 118% there was a propensity to overestimate the cup template by one size with a non-significant yet notable underestimation of one size at 119%. This trend was also noted by Manrique et al. who reported a greater number of one-size-smaller errors when templating at 121% [16]. They concluded the optimal magnification for pre-operative templating to be between 118-119.5%, a best-fit magnification constant also noted in other research [17]. Our study found no significant difference in accuracy between 118% and 119%. 

In contrast, studies templating with ECMs report exact prosthesis matching in 32% of acetabular cups and 43% of femoral stems, improving to 92% and 96% within two sizes of the template respectively [18]. Shaarani et al. too found similar results with exact cup choice in 38% and stem choice in 36%, both increasing to 98% within two sizes [4].

These results suggest ECM calibration risks positioning inconsistency, magnification error [19] and tolerance for excessive size miscalculations [12]. Studies agree the optimal location for ECM placement to be the anatomical centre of rotation (COR) (centre of the femoral head) [20]. Given the infeasibility, there is great debate over the best replication of this position [12,19,21]. Coronal deviation towards the source/away from the plate overestimates the magnification and vice versa [6]. This was exemplified by Ramme et al. who found an ECM AP deviation of 3.5 cm to result in one full cup size change [22]. Similarly, a lateral shift from the midline in the sagittal plane introduced as much as 7% magnification error [23] or 2% for every 17.5cm deviation, dependent on vertical height [19]. These figures explain the significant variation in magnification errors with ECMs and confirm greater accuracy when calibrating at set magnification [6,13].

The effects of BMI on magnification error and subsequent accuracy in implant size estimation are under-reported in the literature. Our results suggest a significant trend in increasing magnification error with increasing BMI, trending towards over-estimating magnification. This finding is echoed by other studies describing a proportional increase in magnification with the distance between the hip and detector plate [19,24], a key confounder in the accuracy of ECM positioning [25]. However, despite the effects on magnification errors, our results prove BMI did not have a significant impact on templating implant size. Few studies investigate the effect of BMI on template accuracy but conclude no significant correlation between template size inaccuracy and BMI of normal, overweight and obese patients [9,26].

We defined accurate acetabular implant choice within two sizes based on previous investigations and our own clinical judgment. Studies debate "accurate sizing" to be within one [12] or two sizes of template [4,26] (2mm difference) in lieu of intra- and post-operative risks. Evidence suggests there is no compromise in the post-operative clinical score when selecting intra-operative implants at one or more size differences from the template [1]. Thus, we believe accurate template predictions to be within two sizes of the prosthesis used. 

We used AP radiographs filmed in the supine position using our trusts’ standardised protocol to address inaccuracies of poorly imaged anatomy [20,27] and positioning effects [3,18]. For other unknowns such as bone quality, posterior acetabular integrity and femoral torsion, we could only hypothesise on their effect. Other imaging modalities, such as 3-D computerised tomography (CT) have demonstrated superior ability in measuring these variables and templating exact implant sizes [20]. These however come at the expense of greater radiation exposure and cost [28], and therefore require strict risk-benefit analysis for justification.

The templating itself may have been influenced by the skill and experience of the consultant surgeon. There is conflict as to how much experience truly affects personal templating ability [29]. However, most studies report little or no difference in templating accuracy amongst all grades of trainee and consultant, presuming they have received adequate training [9,21].

There were several limitations in our study. Data were retrospectively reviewed. To address selection bias, all cases analysed were operated by a single surgeon consecutively and to reduce recall bias, every template and post-operative document was reviewed twice with documentation of errors. The surgeon was not blinded to their template and as such could have been biased towards the final implant choice. However, if different surgeons templated and operated, a difference in opinions/technique could have nullified the template. The operating surgeon was not bound by his template and was at liberty to make intra-operative adaptations.

## Conclusions

In conclusion, this is the first study to directly compare set magnification at two different calibrations. These results confirm that templating THA surgery with a set magnification is a reliable technique for pre-operative planning without the need for calibration equipment. We believe the optimal magnification to be 118-119%; however, we understand this figure would differ between trusts and radiographic systems. Whilst BMI can affect magnification error, this has no significant effect on the accuracy of implanted cups or stems within two sizes and as such, surgeons should feel confident with pre-operative planning via this technique.
